# Role of zinc as an essential microelement for algal growth and concerns about its potential environmental risks

**DOI:** 10.1007/s11356-022-20536-z

**Published:** 2022-05-13

**Authors:** Nagwa I. El-Agawany, Mona I. A. Kaamoush

**Affiliations:** 1 Botany and Microbiology Department, Faculty of Science, Alexandria University, Alexandria, Egypt; 2 Environmental Protection and Crises Management Department, Simulator Complex, Arab Academy for Science, Technology and Maritime Transport (AAST), Alexandria, Egypt

**Keywords:** *Dunaliella tertiolecta*, Photosynthesis pigment, Fatty acids, Protein profile, IR spectra

## Abstract

This work aims to measure the role of zinc as an essential micronutrient for algal growth and the effect of using different concentrations of this heavy metal on growth and essential metabolites of *Dunaliella tertiolecta*. The EC50 obtained was around 15 mg/l. The results obtained proved that lower concentrations of the element increased growth and the content of the measured metabolites (photosynthesis pigments, fatty acids, and protein) but with different responses. The increase in content of these metabolic products with low concentrations of the tested heavy metal may be attributed to inhibition to these metabolites’ export out of cells by heavy metals. The obtained infrared peaks of the major cell constituents of the treated cells revealed the emergence of new peaks and the removal of others, indicating changes in cell constituents due to changing zinc concentrations.

## Introduction

Zinc is a component of various enzymes, including superoxide dismutase, carboxypeptidase, carbonic anhydrase, and a variety of dehydrogenases, and is a necessary mineral nutrient for plant and algal growth (Coleman 1998). Plants with a zinc shortage have a significant reduction in electron transport and phosphorylation. Although zinc is a micronutrient, it is frequently found in high concentrations in algal biomass due to accumulation and sequestration in polyphosphate bodies in cell sectors (Subrahmanyam and Ajay 1996).

The discovery of zinc in many highly purified enzymes has revealed the diversity of its function in protein and carbohydrate metabolism (Hoch and Vallee 1958). Depending on the amount of zinc available, it can function in either a stimulatory or an inhibitory way. Low zinc concentrations aided the growth of certain algae, while high zinc concentrations slowed growth and reduced cell division. Furthermore, several tolerant blue-green algae species and green algae could exist in areas with the highest zinc content. Many studies have found that dead algae acquire much more Zn^2+^ than live algae (Cushing and Watson 1966). Although Zn is an important element for all living organisms’ growth, large quantities of Zn can restrict growth and pose a hazard to ecosystems (Dinesh Kumar et al. 2014).

Many environmental conditions influence zinc toxicity in algae, and the status of the metal is determined by the chemical characteristics of the medium such as pH; particles and complexing agents; presence of other elements and ions such as Ca^2+^, Mg^2+^, P^2+^, C^2+^, and NO_3_^−^ or other metals; sulfur compounds and amino acids; ionic strength; cell concentrations; temperature; salinity; light intensity; aeration of the medium; the exchange reactions between suspended sediments; water; and age of the cells (Jeyachandran and George 1991). ZnO produces toxicological harm to animals after entering the mammalian body through the respiratory and digestive tracts, as well as DNA damage by inducing oxidative stress (Golbamaki et al. 2015).

Determination of the half-maximal effective concentration of heavy metal (EC50) is very important for the estimation of metal toxicity which can use EC50 values of some heavy metals such as Cu, Ni, and Zn determined for growth inhibition of some green algae species to determine their toxicity, for example Cu found to be more toxic to algae than Ni or Zn (Magdaleno et al. 2014; El Agawany et al. 2021). Mechanisms underlying metal tolerance in algae are not fully recognized and may rely on several mechanisms. It was estimated that in the giant cells of *Chara corallina*, most of the intracellular Zn^2+^ was stored in vacuoles (Reid et al. 1996).

De-Filippis and Ziegler (1993) stated that Cyanophyceae are sensitive to Cu, Cd, and Zn metals than other algae tested for photosynthetic activity, through the inhibition of photosystem II and/or reduction in activities of enzymes involved in the fixation of CO_2_. Higher concentrations of Zn^2+^ decreased cell division, movement, total chlorophyll content (De-Filippis et al. 1981a), and carotenoid/chlorophyll ratio (Rai et al. 1981). Microalgae were shown to be a good source of Omega-3 fatty acids, which protect against chronic diseases such as coronary heart disease, diabetes, and cancer (Simonopoulos 1991). The decrease in polyunsaturated fatty acid fractions could be attributed to a decrease in membrane fluidity and permeability (Xu et al. 1998).

## Materials and methods

### Biological material

The biological material employed in this study was *Dunaliella tertiolecta*, a unicellular marine green alga collected from the botany and microbiology department of Alexandria University’s Faculty of Science’s culture collection of algae. Algal culture was checked for axenia by inoculating it on M.H. solid media (Loeblich 1982). Each of the axenic cultures was grown in 50 mL MH medium in 250-mL Erlenmeyer Pyrex-glass flasks under controlled laboratory conditions (temperature at 25 °C ± 3 °C and light at 4000 lx) in a controlled culturing chamber. The temperature inside the growth chamber was kept as possible below 28 °C through periodical ventilation. This temperature was chosen depending on the results of Ginzburg and Ginzburg (1981). Growth determination by cell count of the tested organism was determined by using the hemacytometer slide.

### Preparation of heavy metal stock solutions

Heavy metals (Zn) were chosen for this investigation because of their abundance in wastewater surrounding Alexandria and their discharge into aquatic environments. Stock solutions of the chosen heavy metal were made from salt in double-distilled water and sterilized using 0.2-m nitrocellulose membranes. According to Wong and Pak (1992), a preliminary experiment was carried out utilizing a wide range of metal solutions [ZnCl_2_] to identify the appropriate quantities of these metal salts that the investigated alga could tolerate. The EC50 was found to be around 15 mg/l. Other concentrations (two lower and two higher) had been chosen.

### Chlorophylls and β-carotene estimation

The spectrophotometer method is the simplest method for estimating chlorophyll “a,” “b,” and “β-carotene estimated according to the trichromatic equation of Jeffrey and Humphrey (1975).

### Total lipid extraction and identification

The total lipid content of the tested algal species *Dunaliella tertiolecta* was extracted according to Bligh and Dyer (1959).

### Infrared spectra of tested species cells

Preliminary trials were performed to determine the density of cell suspensions necessary to produce spectra with a good signal. Lugol’s iodine solution was added to pellets of a known volume of algal culture according to Kansiz et al. (1999).

### Protein profile analyses

The protein profile was performed according to the method described by Laemmli (1970). The fresh cell pellets obtained by centrifugation of 10 mL culture were ground in a mortar in the presence of a small volume of 0.5 M Tris–HCl buffer (pH 7.2). The suspension was centrifuged, and the supernatant was concentrated in pre-activated dialysis tubing over a sucrose bed. A few drops of glycerol were added to increase viscosity and 2 to 3 drops of bromophenol blue as an electrophoresis marker. Electrophoresis was carried out in 12% acrylamide running gel and 4% stacking gel, with 0.025 M Tris and 0.19 M glycine buffer at pH 8.3. Further details of the methods used in gel preparation and electrophoresis were described previously by Moore et al. (1958). Electrophoresis was performed at 25 °C in a vertical tank apparatus maintained at 100 V using a constant-voltage power supply, until bromophenol blue tracking dye reached the bottom of the gel. After electrophoresis, gels were stained with 2% Coomassie Brilliant Blue G-250 in water–methanol-acetic acid (4.5:4.5:1) for 4 h at 37 °C; after that, they were destained by agitation in the same solvent without dye, and stored in a fixing solution (7% acetic acid) for at least 30 min at 30 °C and rinsed with deionized distilled water. The gels were then photographed.

### Statistical analysis

The data consisted of the means and standard deviations of at least three independent experiments. The obtained data were statistically examined at 0.05 using the least significant difference (LSD).

## Results and discussion

**Fig. 1:**
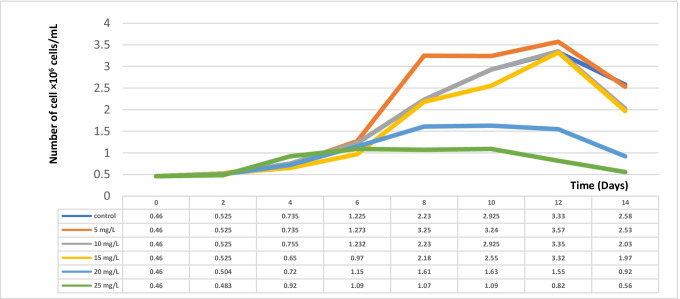
Number of cell × 10^6^ cells/mL of *Dunaliella tertiolecta* cultured for 14 days at different concentrations of Zn^2+^ (mg/l)

The results obtained showed that the EC50 of zinc was 15 mg/L, our result goes with harmony with those obtained by Hou et al. (2018). Using lower concentrations of Zn accelerated the growth of *Dunaliella tertiolecta*, while higher concentrations inhibited the growth, and the degree of inhibition depended on the increase in the concentration of heavy metal. Our results go with those obtained by El-Naggar (1993) who indicated that higher concentrations of Zn reduced the growth of *Chlorella vulgaris*. Also, Ting et al. (2018) observed that high levels of zinc could severely inhibit the growth of *Spirulina platensis*. On the contrary, Inga et al. (2018) observed high accumulation capacity of *Spirulina platensis* to accumulate Zn from industrial effluent and suggested that biomass can be used for the economic treatment of wastewater containing zinc (Fig. 1).

### Estimation of photosynthetic pigments

**Fig. 2:**
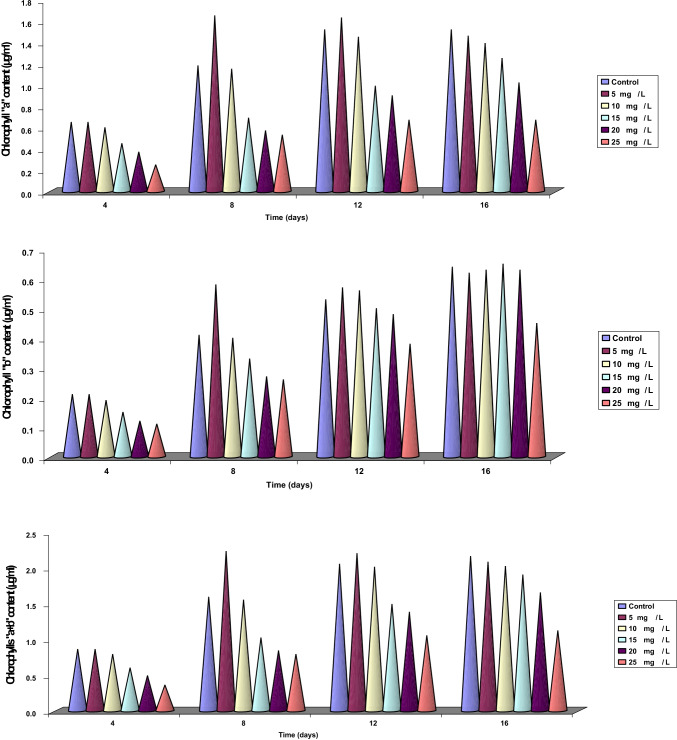
Chlorophyll content a, b, and a + b (µg/mL) of *Dunaliella tertiolecta* cultured for 16 days at different concentrations of Zn^2+^ (mg/L)

Data concerning the effect of different concentrations of Zn^2+^ ions on content of chlorophyll fractions and total chlorophylls in *Dunaliella tertiolecta* after 16 days of culturing were recorded in Table 1 and Fig. 2. These data observed proved that values of chlorophyll content differed according to the Zn^2+^ concentrations used. This impression could be proved from the fact that the ratio between chlorophylls a and b after 4 days of culturing is nearly the same at control and at 5, 10, 15, and 20 mg/L Zn^2+^. After 8 days of culturing, the ratio between chlorophylls a and b is nearly the same for control and for 5 and 10 mg/L Zn^2+^. By increasing the days of culturing, the toxicity of Zn^2+^ was more prominent at higher concentrations (15, 20, and 25 mg/L), where it reached the maximum at 25 mg/L Zn^2+^. At the end of the experiment, the total chlorophyll content at 5 and 25 mg/L decreased by 3.7% and 48.1%, respectively, compared to control.

**Table 1:** Chlorophyll content (μg/mL) of *Dunaliella tertiolecta* after 4, 8, 12, and 16 days of culturing at different concentrations of Zn^2+^ (mg/L)

Time (days)	Parameters	Control	Different concentrations of Zn^2+^ (mg/L)					*F* (*p*)	LSD
			5	10	15	20	25		
4	Chlorophyll a	0.650 ± 0.003 ^a^	0.650 ± 0.001 ^a^	0.600 ± 0.002 ^b^	0.450 ± 0.004 ^c^	0.370 ± 0.002 ^d^	0.25 ± 0.001 ^e^	17,582.143^**^ (< 0.001)	0.002
	Chlorophyll b	0.210 ± 0.001 ^a^	0.210 ± 0.002 ^a^	0.190 ± 0.001 ^b^	0.150 ± 0.002 ^c^	0.120 ± 0.001 ^d^	0.110 ± 0.003 ^e^	2107.059^**^ (< 0.001)	0.002
	**Total**	**0.86**	**0.86**	**0.79**	**0.60**	**0.49**	**0.36**		
	**Ratio (a/b)**	**3.09:1**	**3.09:1**	**3.15:1**	**3.00:1**	**3.08:1**	**2.27:1**		
8	Chlorophyll a	1.180 ± 0.004 ^a^	1.650 ± 0.003 ^b^	1.150 ± 0.003 ^c^	0.690 ± 0.002 ^d^	0.570 ± 0.001 ^e^	0.530 ± 0.004 ^f^	60,551.661^**^ (< 0.001)	0.003
	Chlorophyll b	0.410 ± 0.002 ^a^	0.580 ± 0.004 ^b^	0.400 ± 0.004 ^c^	0.330 ± 0.003 ^d^	0.270 ± 0.003 ^e^	0.260 ± 0.002 ^f^	4008.571^**^ (< 0.001)	0.004
	**Total**	**1.59**	**2.23**	**1.55**	**1.02**	**0.84**	**0.79**		
	**Ratio (a/b)**	**2.87:1**	**2.84:1**	**2.87:1**	**2.09:1**	**2.11:1**	**2.03:1**		
12	Chlorophyll a	1.520 ± 0.003 ^a^	1.630 ± 0.003 ^b^	1.450 ± 0.002 ^c^	0.990 ± 0.003 ^d^	0.900 ± 0.004 ^e^	0.670 ± 0.003 ^f^	44,392.258^**^ (< 0.001)	0.004
	Chlorophyll b	0.530 ± 0.002 ^a^	0.570 ± 0.003 ^b^	0.560 ± 0.003 ^c^	0.500 ± 0.004 ^d^	0.480 ± 0.002 ^e^	0.380 ± 0.003 ^f^	1703.529^**^ (< 0.001)	0.003
	**Total**	**2.05**	**2.20**	**2.01**	**1.49**	**1.38**	**1.05**		
	**Ratio (a/b)**	**2.86:1**	**2.85:1**	**2.58:1**	**1.98:1**	**1.87:1**	**1.76:1**		
16	Chlorophyll a	1.520 ± 0.004 ^a^	1.460 ± 0.002 ^b^	1.390 ± 0.003 ^c^	1.250 ± 0.002 ^d^	1.020 ± 0.003 ^e^	0.670 ± 0.004 ^f^	29,707.619^**^ (< 0.001)	0.004
	Chlorophyll b	0.640 ± 0.002 ^a^	0.620 ± 0.001 ^b^	0.630 ± 0.002 ^c^	0.650 ± 0.001 ^d^	0.630 ± 0.002 ^c^	0.450 ± 0.002 ^e^	7388.571^**^ (< 0.001)	0.002
	**Total**	**2.16**	**2.08**	**2.02**	**1.90**	**1.65**	**1.12**		
	**Ratio (a/b)**	**2.37:1**	**2.35:1**	**2.20:1**	**1.92:1**	**1.61:1**	**1.48:1**

The chlorophyll “a” content as another parameter of growth followed nearly a similar pattern of change to that of growth in response to different concentrations of zinc. Our results go with harmony with those obtained by El-Naggar (1993) who indicated that lower concentrations of zinc induced a significant increase in the different pigment fractions (chlorophyll “a” and chlorophyll “b”) of the green alga *Chlorella vulgaris* and *Scenedesmus bijuga* while higher concentrations of zinc suppressed the level of pigment fractions.

The results were obtained after 4 days of culturing under the effect of 5 and 10 mg/L Zn^2+^ where the content of β-carotene was slightly higher than or equal to the content in the case of control, but after 8 and 12 days of culturing, the content of β-carotene at 5 mg/L Zn^2+^ increased by 40.4% and 7.3%, respectively, compared to control. At 10 mg/L and after 8 days of culturing, the content of β-carotene was equal to control, i.e., 0.622 µg/mL. However, for the remaining concentrations 15, 20, and 25 mg/L Zn^2+^, the content of β-carotene decreased from the beginning to the end of the experiment under the toxic effect of Zn^2+^ ions. The degree of inhibition of photosynthetic pigments depends mainly on metal type, chlorophyll a, chlorophyll b, and carotenoids; the following inhibition orders can be established: Zn > Cu > Ni, Ni > Cu > Zn and Ni > Cu ≥ Zn, respectively, (Alexandra et al 2021).

**Fig. 3:**
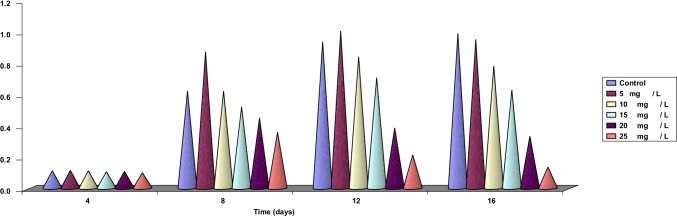
β-Carotene content (µg/mL) of *Dunaliella tertiolecta* cultured for 16 days at different concentrations of Zn^2+^ (mg/L)

Carotenoid content behaved the same way as in the case of chlorophyll a. The total carotenoids at the lowest concentration of Zn^2+^ (5 mg/L) significantly increased gradually over control till the 8th day of culturing. At the other concentrations of Zn^2+^, the content of carotenoids decreased gradually till the end of the experiment, (Fig. 3; Table 2). This result goes in harmony with those obtained by El-Maghrabi (2002) and De-Filippis et al. (1981a). The inhibition in photosynthesis pigment accumulation in response to heavy metal stress might be also a consequence of peroxidation of chloroplast membranes via the increased rate of reactive oxygen species production. Bajguz (2011) indicated that the biosorption of heavy metals by *Cholrella vulgaris* was accompanied by an induction of a variety of biochemical changes and had significant inhibition of growth and photosynthesis. Inhibited biosynthesis of chlorophyll and carotenoids were most frequently observed symptoms of metal toxicity.

**Table 2:** β-Carotene content (μg/mL) of *Dunaliella tertiolecta* after 4, 8, 12, and 16 days at different concentrations of Zn^2+^ (mg/L).

Time (days)	Control	Different concentrations of Zn^2+^ (mg/L)					*F* (*p*)	LSD
		5	10	15	20	25		
4	0.112 ± 0.002 ab	0.115 ± 0.003 b	0.112 ± 0.001 ab	0.106 ± 0.002 c	0.109 ± 0.001 a	0.100 ± 0.002 d	28.800** (< 0.001)	0.002
8	0.622 ± 0.001 a	0.873 ± 0.002 b	0.622 ± 0.007 a	0.520 ± 0.003 c	0.449 ± 0.003 d	0.359 ± 0.004 e	6532.153** (< 0.001)	0.004
12	0.937 ± 0.003 a	1.005 ± 0.001 b	0.841 ± 0.002 c	0.707 ± 0.002 d	0.386 ± 0.002 e	0.214 ± 0.001 f	95,096.716** (< 0.001)	0.002
16	0.989 ± 0.003 a	0.952 ± 0.003 b	0.782 ± 0.002 c	0.629 ± 0.003 d	0.333 ± 0.002 e	0.136 ± 0.003 f	45,988.813** (< 0.001)	0.003

Different lowercase letters denote significance. Data are expressed in mean ± SD.

*F* (*p*) *F*-test (ANOVA) and its significance between groups, *LSD* least significance difference at 0.05.

*Statistically significant at *p* ≤ 0.05; **statistically significant at *p* ≤ 0.01.

### Fatty acid fractions

It is a well-known fact that lipid production usually differed between genera, species, and strains of microalgae (Johansen et al. 1987). However, in healthy phytoplankton, total lipid fractions range from less than 1% to more than 40% of dry weight (Dubinsky et al. 1978).

**Fig. 4:**
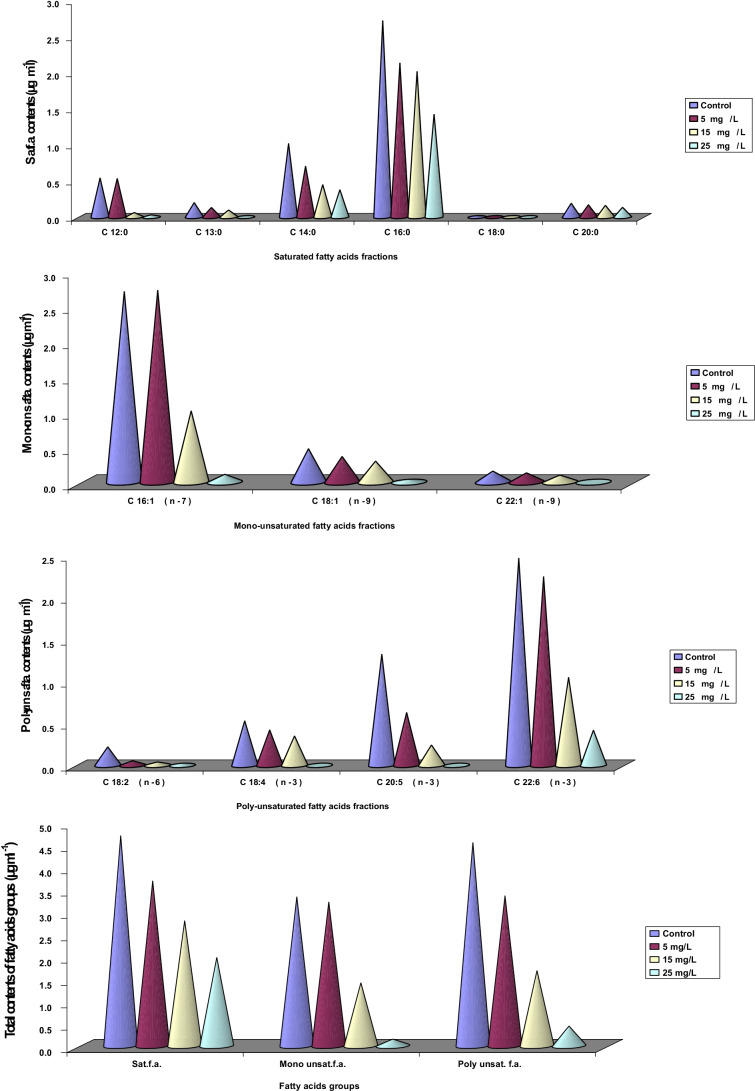
Fatty acid fractions (mg/ml cultured) for  *Dunaliella tertiolecta* cells cultured at different concentrations of Zn^2+^ 5, 15, and 25 mg/L after 14 days

Taking into consideration the effect of the tested three different concentrations of Zn^2+^ (5, 15, and 25 mg/L) on the content of the three groups of fatty acids (saturated, mono-unsaturated, and poly-unsaturated fatty acids), it is clear from the data recorded in Table 3 and graphed in Fig. 4 that at 5 mg/L Zn^2+^ the percent of decrease in the total content of saturated fatty acids reached nearly 21.53% compared to control. It is also clear that C18 which was present in the control disappeared completely at 5, 15, and 25 mg/L Zn^2+^. The synthesis of saturated fatty acids at 15 and 25 mg/L Zn^2+^ was greatly affected by these two concentrations. The effect was more prominent at 25 mg/L than at 15 mg/L Zn^2+^. The percent of decrease reached 40.44 and 57.90% compared to control. Although saturated fatty acids are greatly affected by 5 mg/L Zn^2+^, the mono-unsaturated fatty acids at the same concentration are slightly affected. The percent of decrease in the content of mono-unsaturated fatty acids at 5 mg/L Zn^2+^ reached nearly 3.5% compared to control. On the contrary, at 15 and 25 mg/L Zn^2+^ the percent of decrease in the content of this group of fatty acids reached nearly 57.64% and 95.41%, respectively, compared to control. It is also clear that C22:1 at 25 mg/L Zn^2+^ disappeared completely indicating that this fatty acid was more sensitive to higher concentrations of Zn^2+^. The third group of fatty acid (i.e., poly-unsaturated fatty acid) was the only group that was greatly affected by Zn^2+^ ions specially at 15 and 25 mg/L Zn^2+^. The percentage of decrease at 5, 15, and 25 mg/L Zn^2+^ reached nearly 26.15%, 62.96%, and 90.24%, respectively, compared to control. At 25 mg/L Zn^2+^, the poly-unsaturated fatty acid fractions C18:4 and C20:5 disappeared completely. However, the ratio between the total content of fatty acid at control and the three concentrations of Zn^2+^ reached 10.24: 7.56: 3.79: 1, respectively.

**Table 3:** Effect of different concentrations of Zn^2+^ (5, 15, 25 mg/L) on the content of fatty acid fractions (µg mL^−1^) of *Dunaliella tertiolecta* cultured on MH basal medium for 14 days

Fatty acids		Control	Different concentrations of Zn^2+^		
			5 mg/L	15 mg/L	25 mg/L
Saturated fatty acids	C12:0	0.545	0.537	0.065	0.032
	C13:0	0.202	0.134	0.100	_
	C14:0	1.021	0.708	0.451	0.381
	C16:0	2.724	2.138	2.019	1.429
	C18:0	0.016	_	_	_
	C20:0	0.193	0.172	0.165	0.137
Total		**4.701**	**3.689**	**2.800**	**1.979**
% of decrease		**_**	**21.53**	**40.44**	**57.90**
Mono unsaturated fatty acids	C16:1 (n-7)	2.702	2.722	1.011	0.113
	C18:1 (n-9)	0.476	0.365	0.300	0.040
	C22:1 (n-9)	0.158	0.132	0.102	_
Total		**3.336**	**3.219**	**1.413**	**0.153**
% of decrease		**_**	**3.51**	**57.64**	**95.41**
Poly unsaturated fatty acids	C18:2 (n-6)	0.221	0.055	0.041	0.025
	C18:4 (n-3)	0.532	0.423	0.351	_
	C20:5 (n-3)	1.325	0.630	0.243	_
	C22:6 (n-3)	2.469	2.250	1.049	0.419
Total		4.547	3.358	1.684	0.444
% of decrease		_	26.15	62.96	90.24
Total content		12.584	10.266	5.897	2.576
% of total content		_	18.42	53.14	79.53

The essentiality of zinc, which is thought to be universally required by algae, can be linked to the stimulation that only manifested at low concentrations of Zn^2+^ in growth, chlorophylls “a” and “b,” and fatty acid content of the investigated organism. It is vital for carbonic anhydrase enzymes which catalyze the dehydration of carbonic acid and participate in the elimination and incorporation of carbon dioxide. So, it could be concluded from our results that zinc is an essential element in photosynthesis and carbohydrate metabolism. This result is in harmony with those obtained by Ajay and Subrahmanyam (1996). However, there are many tolerant species of cyanobacteria and green algae which could tolerate and survive at high Zn^2+^ concentrations (Engel et al. 1981). Furthermore, some heavy metals have been discovered to contribute to lipid synthesis in microalgae, for example Mg^2+^ could enhance the lipid accumulation (Ren et al. 2014).

Regarding fatty acid content under the effect of lower concentrations of Zn^2+^ in the culture media of *Dunaliella tertiolecta*, the results show that these lower concentrations stimulated the content of this metabolic parameter, while higher concentrations of Zn^2+^ inhibited its biosynthesis. The increase in content of this metabolic products with low concentrations of heavy metals may be attributed to inhibition to this metabolite export out of cells by heavy metals as reported by De-Filippis et al. (1981a, b). Similar observations were reported also by El-Naggar (1993). However, it is well known that accumulation of metabolic products at low heavy metal concentrations may be one of the ways through which the algae can abolish their toxic effect (Rai et al. 1991). Dowidar (1983) mentioned that saturated fatty acids were more dominant than unsaturated ones under stress conditions. The total content of the three types of fatty acids reduced as the element’s concentration was increased. This decrease was found to be very highly significant, i.e., the toxic effect increased with increasing element concentration. El-Sheikh et al. (1999) recorded that toxicity of the element was concentration dependent. The toxic effect of all groups of fatty acids ranged from highly significant at low concentrations to very highly significant at higher ones. At higher concentrations, the synthesis of all groups of fatty acids was inhibited by the three concentrations used, but the degree of inhibition was more prominent at higher concentrations especially in the content of polyunsaturated fatty acids.

### Infrared spectra

**Fig. 5:**
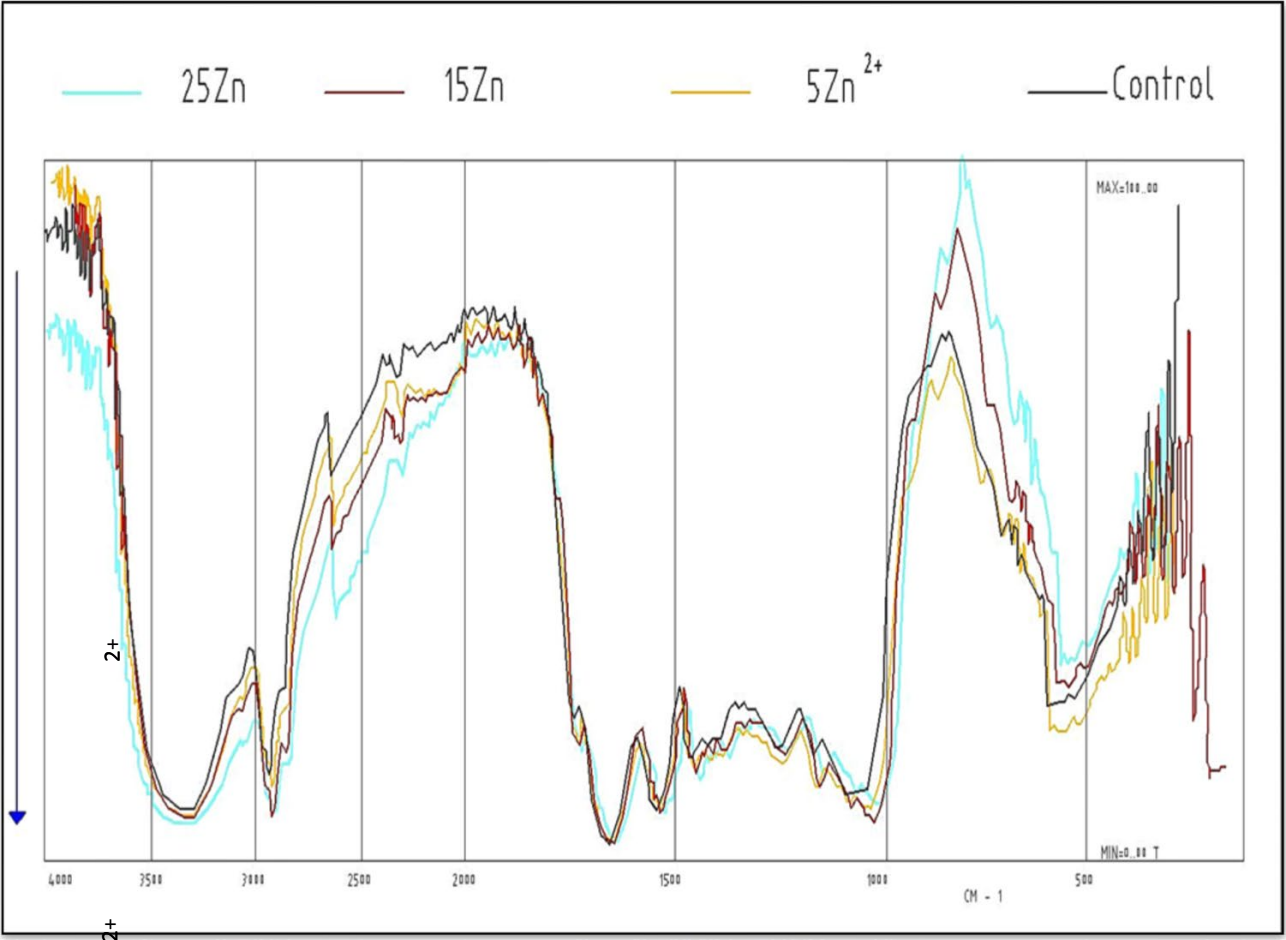
IR spectra of the total cell constituents of the studied *Dunaliella tertiolecta* cultured under the effect of different concentrations of Zn^2^

The results reflected the total biochemical composition of the algal cells. The obtained results based on cell spectra of *Dunaliella* species show the band assignments which are based on the studies of whole-cell organelles and macromolecules at the region from 4000 to 250 cm^−1^. The obtained IR spectra and number of peaks that appeared new or disappeared among the major cell constituents of *Dunaliella tertiolecta* cultured for 8 days under the effect of 5, 15, and 25 mg/L were recorded as shown in Table 4 and graphed in Fig. 5.

**Table 4:** Number of frequency distribution of IR spectroscopy of total cell constituents of *Dunaliella tertiolecta* cultured for 8 days under the effect of different Zn^2+^ concentrations

Frequency	Control	5 mg/L Zn^2+^				15 mg/L Zn^2+^				25 mg/L Zn^2+^			
		Present	Absent	New	Total	Present	Absent	New	Total	Present	Absent	New	Total
4000–3640	17	15	2	4	19	14	3	4	18	16	1	3	19
3000–2010	10	6	4	4	10	8	2	5	13	7	3	4	11
2000–1720	10	7	3	2	9	10	–	2	12	7	3	4	11
~ 1650	1	–	1	1	1	1	–	1	2	1	–	–	1
~ 1540	1	1	–	1	2	1	–	1	2	1	–	2	3
1450–1410	2	2	–	1	3	2	–	1	3	2	–	2	4
~ 1335	1	1	–	1	2	1	–	1	2	1	–	1	2
1240–1160	2	2	–	–	2	1	1	1	2	2	–	–	2
1080–1000	1	1	–	1	2	1	–	1	2	1	–	1	2
700–250	14	13	1	6	19	12	2	5	17	13	1	5	18
Total	**59**	**48**	**11**	**21**	**69**	**51**	**8**	**22**	**73**	**51**	**8**	**22**	**73**

At 5 mg/L Zn^2+^, the total peaks obtained in the case of control (59 peaks) increased to 69 peaks. At a frequency 4000–3640 cm^−1^, the organism synthesized 19 compounds, 4 of which were synthesized new and 15 remained unchanged and 2 disappeared. At frequencies 3000–2010 cm^−1^, 4 peaks disappeared while 4 peaks appeared new. The only frequencies at which the peaks remained without any change are from 1240 to 1160 cm^−1^. At frequencies from 1540 to 700 cm^−1^, the number of peaks present in the control remained unchanged, but new peaks appeared in addition to the original ones. Table 4 explains these facts which show the number of peaks that are present and absent and those that newly appeared at the different frequencies recorded. At 15 mg/L Zn^2+^, the number of peaks increased from 59 in control to 73 peaks. This means that more compounds were assimilated compared to control. In the region of vas C-H of methylene groups (at frequency 4000–3640 cm^−1^), the organism synthesized 18 compounds, 4 of which were new, while 14 compounds remained as in control. The results of IR obtained at 25 mg/L Zn^2+^ gave 73 peaks, 6 of which disappeared and 22 peaks appeared new in addition to 51 peaks that only remained unchanged. Our results go with harmony with those obtained by Kansiz et al. (1999) and El Agawany (2008).

Infrared spectroscopy cleared that the treated organism with the three used concentrations of the tested metals showed usually other peaks resulting new, either from the changes in the positions of some side chains of the same compounds or from the disappearance of some compounds. This could be proved from the results obtained which revealed that toxic effects depend on the type of the element and its concentration (El-Sheikh et al. 1999).

### Total soluble protein profile

**Fig. 6:**
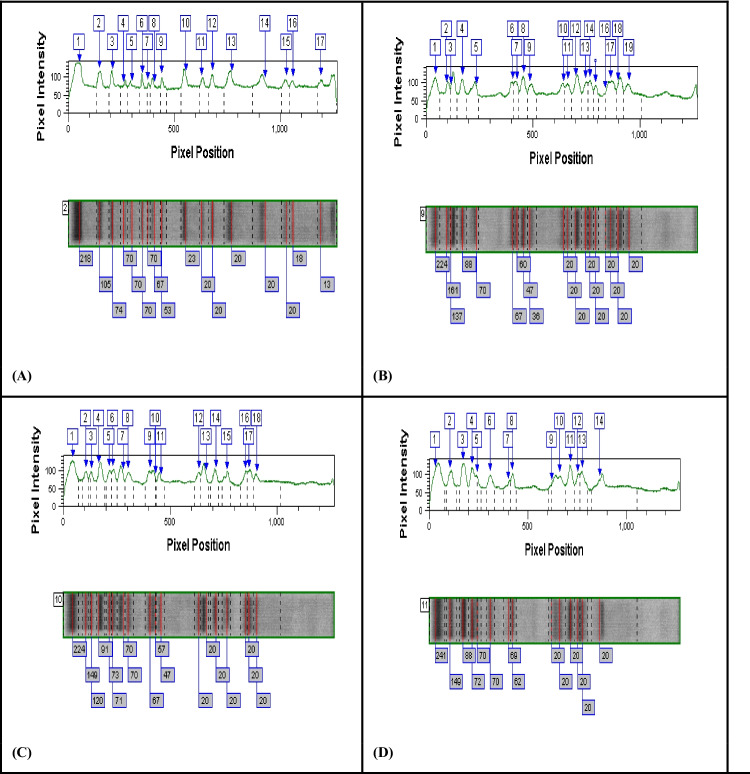
Electropherogram showing the results of scanning of protein profile bands of *Dunaliella tertiolecta* cells cultured **a** on basal medium (control) and under the effect of different concentrations of Zn^2+^ **b** 5 mg/L, **c** 15 mg/L, and **d** 25 mg/L

It is clear from the results that the obtained bands of the protein profile were distributed throughout the gel plate. Some bands are cathodic, and others are anodic, but most of the bands have cathodic anodic symmetry. The sum of the bands that appeared on the gel plate and confirmed by scanning using the band peaks was 33 bands. Some of these bands were common in the control and the treated organism; others were common only in the treated organism under the effect of the three different concentrations of the three tested elements. However, in nearly all the lanes most of the bands appeared in the region between 13 and 241 kDa. A glimpse at the number of bands obtained for 17 control-only bands were observed, while their number increased or decreased depending on the type and concentration of the metal ion. This idea could be clearly confirmed in the protein profile shown in Table 5 and graphed in Fig. 6. This table clearly shows the total number of bands that appeared (the unchanged, the disappeared, and the newly formed bands). It is clear also in Fig. 6 that at 25 mg/L of Zn^2+^, the number of the newly formed bands decreased. Also, the sum of total bands increased by increasing the concentration of the element at 5 mg/L and 15 mg/L Zn^2+^. The percentage of increase in number of bands was found to depend on the toxicity of the element. The number of the newly formed bands at 15 mg/L was 4, 8, and 11 for Zn^2+^. It is clear also that at 25 mg/L, the organism greatly suffered from the toxic effect of the high concentration of the element. Hou et al. (2018) proved that Zn^2+^ can be rapidly released into algal cells when ZnO NPs were absorbed by lysosomes, and its products lead to produce a large number of free radicals which directly bind to the intracellular proteins.

**Table 5:** Soluble protein profile pattern bands showing sum, unchanged, disappeared, and newly formed bands at different concentrations of Zn^2+^

Element	Control	Zn^2+^ (mg/L)		
Concentration		5	15	25
Sum of bands	**17**	19	18	14
Unchanged bands		8	10	8
Disappeared bands		9	7	9
Newly formed bands		11	8	6

Sinha and Hader (1996) found that *Anabaena* species cultured at stress conditions did not show any changes in the protein pattern. On the other hand, Fulda et al. (1999) found that the composition of periplasmic proteins obtained from cells of *Synechocystis* species grown under stress showed clear differences. Also, Maha (2003) reported that two new bands appeared in *Nostoc linckia* cultivated under stress conditions that were not present in the control culture. Hoyos and Zhang (2000) are in agreement with those obtained results, who found that reversible protein phosphorylation/dephosphorylation plays an important role in signaling the plant adaptive response to stress. However, Salah El-Din (1994) found that most of the algal species have similar physiological functions which are related to biosynthesis or biodegradation of the same macromolecules. On the other hand, Dhargattar (1986) concluded that changes in the biochemical composition of some green algae grown under stress conditions may be related to the chemical and morphological changes associated with the various metabolic processes of the algae. This conclusion seems to explain the different changes of the amount of total soluble protein bands for *Dunaliella tertiolecta*. Awatif (2002) reported that the tolerance of an organism to stress conditions could be achieved through synthesis or accumulation of new protein.

## Conclusion

The effect of the different concentrations of Zn^2+^ on growth, photosynthetic pigments, and synthesis of fatty acids of *Dunaliella tertiolecta* proved that lower concentrations of the element increased the content of the measured metabolites but with different responses. The increase in content of these metabolic products with low concentrations of the tested heavy metal may be attributed to inhibition to these metabolites’ export out of cells by heavy metals. On the other hand, inhibition in their accumulation induced by higher concentrations of heavy metals may be attributed to the toxic action of these heavy metals on the enzymatic reactions responsible for the biosynthesis of these metabolites. Regarding content of fatty acids by increasing the concentration of the element, the total content of the three fatty acid groups decreased. The toxic effect of Zn^+2^ was more prominent in the case of mono-unsaturated and poly-5-unsaturated fatty acids than in the case of saturated ones. The obtained infrared peaks of the major cell constituents of the treated *Dunaliella tertiolecta* showed the appearance of new peaks and the disappearance of others which indicates changes in cell constituents due to the presence of different concentrations of zinc element. It is clear from the results obtained from the protein profile that the percentage of increase in the number of bands was found to depend on the toxicity of the element. It is clear also that at 25 mg/L, the organism greatly suffered from the toxic effect of the high concentration of the element.

## Data Availability

All data generated or analyzed during this study are included and available in this article.
